# Pylorus Preserving Pancreaticoduodenectomy for Peri-Ampullary Carcinoma, Is It a Good Option?

**DOI:** 10.4103/1319-3767.61231

**Published:** 2010-04

**Authors:** Faisal Alsaif

**Affiliations:** Department of Surgery, College of Medicine, King Saud University, Riyadh, Saudi Arabia

**Keywords:** Pancreaticoduodenectomy, peri-ampullary carcinoma, pylorus preserving

## Abstract

Pancreaticoduodenectomy (PD) is the standard surgical treatment for resectable peri-ampullary tumors. It can be performed with or without pylorus preservation. Many surgeons have a negative opinion of pylorus preserving PD (PPPD) and consider it an inferior operation, especially from an oncological point of view. This article reviews the various aspects of PD in the context of operative factors like blood loss and operation time, complications such as delayed gastric emptying and anastomotic leaks, and the impact on quality of life and survival. We aim to show that PPPD is at least as good as classic PD, if not better in some aspects.

Pancreaticoduodenectomy (PD) remains the standard surgical treatment for resectable peri-ampullary tumors. The first PD operation was reported by Codavilli in 1898 in a patient with an epithelioma of the pancreas, but the patient died from cachexia on the 21^st^ post-operative day.[1] In 1946, Whipple described a one-stage PD in which the pylorus was resected.[2] The first report of pylorus preserving PD (PPPD) was by Watson in 1944 for ampullary carcinoma[3] but it did not gain popularity at that time.

In both the classic PD and PPPD, the head of pancreas, duodenum, and distal bile duct are resected. The main difference is that in classic PD, the gastric antrum and pylorus are resected with the creation of a gastro-jejunostomy, while in PPPD, the gastric antrum and pylorus are preserved and the line of resection is through the first part of duodenum and a duodeno-jejunostomy is performed [Figure 1 a and b].

**Figure 1:**
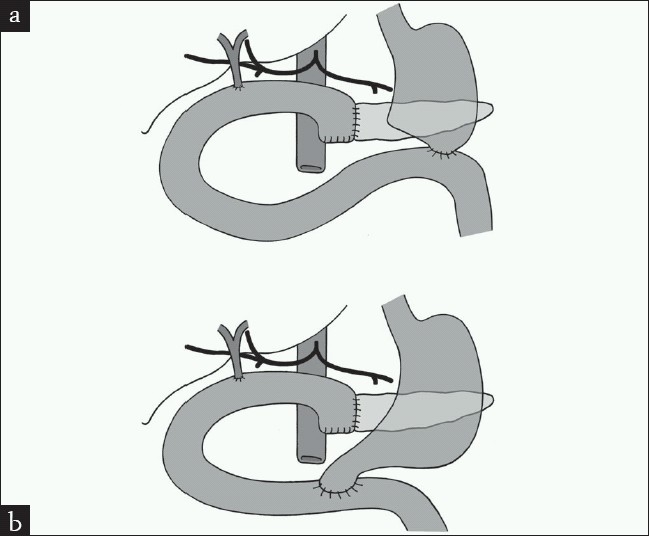
Pancreaticoduodenectomy, (a) classic, (b) pylorus preserving

Traverso and Longmire reintroduced the concept of PPPD for benign peri-ampullary lesions in 1978 as they thought it would decrease the incidence of post-gastrectomy complications.[4] In 1980, they published their experience in PPPD for malignant lesions which included 18 patients with peri-ampullary, duodenal, and pancreatic carcinomas with encouraging results of normal gastric emptying and acidity.[5] Since, PPPD has been applied widely to patients with peri-ampullary lesions, benign, or malignant.

In spite of the reported good outcomes of PPPD, many surgeons still question the benefit of this procedure especially the reported high incidence of delayed gastric emptying and, more importantly, the negative impact that pylorus preservation has on tumor clearance, recurrence, and long-term survival.

We will try in this review article to answer the question of the safety of PPPD as compared to classic PD (CPD) in terms of operative factors, peri-operative complications, tumor recurrence, survival, and long-term quality of life.

## OPERATIVE FACTORS

### Operating time

Sugiyama in 2000 compared 10 patients with PPPD to 14 patients with CPD.[6] Although there was a trend toward a shorter operative time in the PPPD group, it did not reach statistical significance and that was due to a low volume study. A large, multicentre, randomized, controlled trial of 170 patients comparing PPPD with PD also had found no significant difference in the operating time.[7] In a meta-analysis, Traverso had confirmed the previous observation where there had been a trend toward a shorter operating time in PPPD but also not statistically significant.[8]

Two large volume retrospective studies have looked at the operating time difference between PPPD and CPD and it had been clear that the PPPD operating time was significantly shorter than that of CPD.[9,10] That observation has been further supported by a meta-analysis by Karanicolas in 2006 and has found that PPPD was 72 min shorter than PD.[11] A more recent meta-analysis[12] has also shown that PPPD was 41.3 min shorter.

### Blood loss and a need for blood transfusion

Several reports have indicated no significant difference in intra-operative blood loss and blood transfusion between PPPD and PD.[6–8] In a meta-analysis, however, although there has been no significant difference in blood loss, more patients in the PD group have required blood transfusions.[12]

Other studies with a larger patient volume, on the other hand, have shown significantly less blood loss and blood transfusions in the PPPD group[9–11] that could be partly due to the fact that there is less dissection in PPPD. This observation is very important, as blood transfusions in pancreatic cancer have been associated with a decreased survival rate.[13] So if an operative procedure results in less blood loss it should translate into a longer survival.

### Operative mortality

In retrospective analyses, peri-operative mortality has been similar in PPPD and PD groups.[9,10,14] Two meta-analysis studies have shown a trend toward lower peri-operative mortality in the PPPD group.[11,12]

A randomized controlled trial comparing 13 patients with CPD to 14 patients with PPPD has shown no significant difference in mortality (15.4% and 28.6%, respectively, *P*-value 0.65) but these are very high mortality rates for any pancreaticoduodenectomy in comparison to the widely reported 3% in most studies.[15] In a multicentre, randomized, controlled trial involving 170 patients, mortality has been 7% in the CPD group vs. 3% in the PPPD group (*P*-value 0.27)[7]

## POST-OPERATIVE COMPLICATIONS

### Delayed gastric emptying

DGE is probably one of the most studied complications following any type of pancreaticoduodenectomy. There has always been the thought that pylorus preservation would increase the chance of DGE. In a large series from Japan including 1066 patients who underwent PPPD, the incidence of DGE was 46%,[16] which supported the idea of higher DGE with PPPD. A small volume, randomized controlled trial has shown DGE to be 15% in PD vs. 64% in the PPPD group (*P*-value 0.2).[15]

On the other hand, several other studies have not shown the same observation. A retrospective analysis of 113 patients has shown no significant difference in DGE but half of PPPD patients with DGE had co-existing intra-abdominal complications which could have attributed to DGE.[14]

Two retrospective studies have shown no significant difference in DGE between the two groups.[6,9] This was also confirmed in a multicentre, randomized, controlled trial.[13] A retrospective analysis of 239 patients showed that DGE in the CPD group was double that of the PPPD group (6 vs. 13%), but there was a higher percentage of T4 and more extensive resections in the CPD group.[10]

Several meta-analysis studies have also shown that DGE is not higher in the PPPD group.[8,11,12,17]

It seems that DGE is not increased by preservation of the pylorus rather, by other factors including postoperative complications especially intra-abdominal collections. The presence of portal venous hypertension and preoperative cholangitis also increases the chance of post-operative DGE.[18,19]

Shan[22] has made a distinction between subjective DGE and objective DGE as measured by cholescintography and has concluded that although subjective DGE was higher in the PPPD group, objective DGE was similar between the CPD and PPPD groups. He has proposed that loss of the distal stomach mechanoreceptors in the CPD group reduces the patient's sensation of subjective DGE.

Additionally, Kim[23] proposed that pylorospasm could be a cause of DGE in PPPD and has shown a decrease incidence of DGE with the addition of pyloromyotomy. On the other hand, other studies have shown that abnormal gastric motility post surgery is the main cause of DGE regardless of the type of reconstruction.[24,25]

Several methods have been tried to further decrease the incidence of DGE in PPPD. The drug erythromycin has been shown to increase contractility of the stomach and decrease the incidence of DGE.[26,27] On the other hand, somatostatin which is sometimes used to decrease the severity of pancreatic anastomosis leak increases the chance of DGE by more than 3-fold.[28]

An interesting observation was that the use of ante-colic doudeno-jejunostomy as opposed to a retro-colic reconstruction in PPPD decreased the incidence of DGE.[19–21]

### Anastomotic leak

Anastomotic leak, especially from pancreatico-jejunostomy (PJ), is the main factor for morbidity post-PD. A review of 1066 PPPDs in Japan has revealed a leak rate of 16%.[16] In a randomized, controlled trial and two meta-analyses, there has been no difference between CPD and PPPD in terms of PJ leak rate.[11,12,15] Tani[29] has shown that the Traveso-type construction (Duodeno-jejunostomy (DJ) distal to PJ) has a lower leak rate than the Billroth I type reconstruction (DJ proximal to PJ).

### Intestinal acidity and anastomotic ulceration

Not performing an antrectomy could, in theory, result in higher intra-gastric and intestinal pH in the PPPD patient in comparison to the CPD patient. Geenen *et al.* [30] has found that intra-gastric and intestinal pH was not reduced in the PPPD patient but in fact intestinal pH was increased.

Marginal ulceration in PPPD was increased with the use of Roux-en-Y jejuna loop which is not exposed to the diluted effect of pancreatic juice.[31]

### Hospital stay

Usually, the reason for a prolonged hospital stay is either anastomotic leak or DGE. As indicated above, there is no significant difference between CPD and PPPD in terms of leak rate or DGE, so hospital stay should be no different.

Several retrospective, prospective and meta-analysis studies failed to indicate that PPPD causes an increase in hospital stay.[8,12,14,15] In fact, one meta-analysis and one retrospective study showed a trend toward a shorter hospital stay with PPPD[9,11]

## TUMOR RECURRENCE AND LONG-TERM SURVIVAL

The adequacy of PPPD as a cancer operation has always been questionable especially after Sharp and his colleagues had reported three cases of PPPD where the duodenal resection margin was positive for carcinoma.[32] That observation, however, was not supported by other studies. In a retrospective study in 1993, pathological examination of all positive margins in PPPD for peri-ampullary carcinoma has shown that the most common site for a positive margin was peri-pancreatic soft tissue followed by the pancreatic resection line and then the bile duct resection line and no duodenal-positive margin was identified.[33]

The adequacy of resection was further supported by a Japanese group who has found that the number of lymph nodes retrieved was equal in PPPD and total pancreatectomy, which is even more radical than the classic CPD. The number of positive lymph nodes was also no different.[8]

One paper has shown that diffuse peritoneal seeding recurrence was higher in the PPPD group, while liver metastasis and retroperitoneal recurrence were higher in the CPD group.[34] Out of nine patients in the PPPD group who developed retroperitoneal recurrence, six developed obstruction of the first jejuna loop requiring bypass surgery to relieve the obstruction; therefore, retroperitoneal passage of jejuna loop should be avoided. In another study, however, there was no difference in type of recurrence.[35] Both studies revealed no difference in rate of recurrence in general between PPPD and CPD.

Long-term survival was studied extensively as it is the main measure of cancer surgery efficacy. Several retrospective studies have shown that the type of resection does not influence survival.[6,10,14,34,35] Two randomized, controlled trials have also shown no difference in survival.[7,15] A recent meta-analysis has found that a 5 year survival rate was higher in the PPPD group when all tumors where included (*P*-value 0.002), but in the peri-ampullary tumor group there has been no difference in the survival rate.[12]

## QUALITY OF LIFE

The main reason for adopting PPPD was to reserve the whole stomach and to improve digestive function. Post-operative weight gain was comparable between the PPPD and CPD groups[12,13] but patients in the PPPD group reported better gastrointestinal quality of life in terms of appetite, nausea, and diarrhea and an earlier return to work.[12] Although weight gain was comparable, pre-operative weight was reached faster in PPPD patients and they exhibited a better mixture of food with bile.[10,36]

Hyperalimintation for malnutrition was less and serum albumin was higher 6 months following surgery in the PPPD group.[6]

In patients receiving post-operative chemotherapy, a significant increase in body weight was seen with the preservation of the pylorus.[14]

## CONCLUSION

PPPD in comparison to CPD for peri-ampullary carcinoma is at least as effective in terms of peri-operative morbidity or mortality, tumor recurrence, or long-term survival. It may have some advantages in terms of a shorter operating time, less blood loss, fewer blood transfusions, and a better quality of life.

Therefore, PPPD (a well established procedure) remains a good option for any patient with peri-ampullary carcinoma except if the first part of the duodenum or pylorus is grossly involved with tumor.
